# Clinical-grade plant-made nanomaterials: from process design to the construction of a manufacturing facility

**DOI:** 10.3389/fpls.2025.1724810

**Published:** 2026-01-02

**Authors:** Denise Pivotto, Anthony Rosa, Aya Maged Elsheikh, Elisa Gecchele, Roberta Zampieri, Alessia Raneri, Valentina Garonzi, Linda Avesani

**Affiliations:** 1 Department of Biotechnology, University of Verona, Verona, Italy; 2 Diamante SB Srl, Verona, Italy

**Keywords:** nanobiotechnology, peptide Liprin (pLip), plant molecular farming, rheumatoid arthritis, TBSV, tolerance induction, viral nanoparticles

## Abstract

Plant-made nanomaterials are proteinaceous elements that are emerging as multi-purpose and versatile tools in the therapeutic landscape. In the context of autoimmune diseases, Tomato Bushy Stunt Virus (TBSV) has been previously explored as a platform for inducing immune tolerance by displaying disease-specific immunodominant peptides—offering a potential path toward disease remission. In this study, we developed a dedicated facility and a Good Manufacturing-compliant Process for producing TBSV-based nanoparticles engineered to display peptides relevant to specific autoimmune disorders. Data collected from multiple non-consecutive pilot-scale production batches were used to build a simplified techno-economic model of the process. The process is readily scalable and offers opportunities for further improvements, supporting the potential to meet market demands for early-stage therapeutic interventions in autoimmune diseases. Additionally, a preliminary Environmental, Health, and Safety (EHS) assessment of the process showed a highly favorable environmental output index and minimal associated risks, reinforcing the platform’s sustainability. These results support the viability of plant-based manufacturing for therapeutic nanomaterials and highlight TBSV’s potential as a novel platform for tolerance-inducing treatments in autoimmune diseases.

## Introduction

Plant Molecular Farming, which includes the production of recombinant proteins and nanomaterials using plant biotechnology, could provide a steep change in improving health outcome, especially in developing countries and in pandemic contexts, due to its scalability, safety and speed.

The first current Good Manufacturing Process (cGMP) facility producing Plant-Made Pharmaceuticals was designed by Large Scale Biology Corporation (LSBC) in Owensboro, KY, USA (now Kentucky BioProcessing) and opened in the year 1999, using the plant-virus transient expression system Geneware^®^ (Pogue et al., 2002, 2010). Ever since, PMF has developed as a safe, easily scalable and cost-effective technology to produce biopharmaceuticals, when compared to other cell-based systems. Canadian former enterprise Medicago, now acquired by Aramis Biotechnology, was one of the pioneers in the industry. Founded in 1999, its focus was the production of Virus-Like Particles (VLPs) as vaccines, reaching phase III clinical studies with their product Covifenz^®^, a vaccine candidate for the treatment of SARS-CoV2, before announcing their sudden closure in 2023.

Within this continuous framework and thanks to ongoing efforts in basic research, various emerging applications now rely on plant-made nanomaterials, primarily derived from or inspired by viral structures, as promising tools for addressing multiple human diseases. These materials represent a natural progression in the application of plant biotechnology to create innovative solutions for a range of human diseases.

Viruses are excellent examples of naturally occurring nanoparticles that can serve as ideal platforms for the development of new biomedical applications. Plant viruses, in general, are non-enveloped structures, and they can assume a spherical/icosahedral or filamentous/tubular shape. Plant virus capsids are formed through the self-assembly of repeating protein subunits, providing a high degree of multivalency. Their repetitive structure support their function as adjuvants and provide an ideal scaffold for the display of peptides and delivery of drugs (Chung et al., 2020; Santoni et al., 2020). As plant viruses they also have an inherent safety profile in humans.

Tomato Bushy Stunt Virus (TBSV) is a virus of the Tombusvirus family, characterized by an icosahedral capsid of ~ 32 nm in diameter made up of 180 subunits of the coat protein (CP). TBSV Nanoparticles (NPs) can either encapsulate or present small molecules and polypeptides on the surface (Grasso et al., 2013) and are not toxic or teratogenic (Blandino et al., 2015). When intravenously injected into mice, these NPs do not induce alterations of tissues/organs (Lico et al., 2016).

We recently demonstrated the potential of plant-made nanoparticles for preventing and curing autoimmune diseases such as Type 1 Diabetes (T1D) and Rheumatoid Arthritis (RA) (Zampieri et al., 2020). Indeed, the immense potential of nanomaterials as tolerogenic agents to be used in the context of diverse immunological diseases such as autoimmune diseases, allergies and transplants has been extensively demonstrated (Kusumoputro et al., 2023); however, plant viruses have never before been employed for such applications prior to our study.

For RA, we demonstrated that the use of plant-made nanoparticles was able to induce regulatory T cells by diverting the autoimmune response to a tolerogenic setting that reversed the clinical score of the disease with outcomes comparable to golden standard treatments based on immunosuppressants (Zampieri et al., 2020). We used the TBSV genome as a vector of expression and exploited the TBSV Coat Protein for the display of the peptide Liprin (pLip) on the external surface of the capsid.

TBSV NPs manufacturing in plants relies on the use of transient viral expression in *Nicotiana benthamiana*, widely used as a bioreactor for the production of biopharmaceuticals, due to its versatility and susceptibility to plant pathogens. Besides acting as vectors that regulate gene expression (Bally et al., 2018), plant viruses can be genetically engineered to incorporate non-native peptides into their CPs. The modified viruses are then propagated in host plant leaves, which are subsequently harvested and processed for purification (Pogue et al., 2002).

In this sense, once harvested after infection, the plant leaves can be processed to obtain a purified virus that presents a target peptide. This whole NP represents the final Active Pharmaceutical Ingredient (API). Being a plant pathogen, the use of these NPs for therapeutical purposes is entirely safe for humans, due to the absence of specific receptors for viral recognition and penetration into host cells (Nikitin et al., 2016).

While there is currently no clinically approved plant-based nanomedicine in the market, several are undergoing preclinical development while some systems are poised to enter translational development. Given our promising preclinical evidence, we made a concerted effort to spin-out the project from an academic setting by establishing a GMP-grade manufacturing facility, enabling the production of NPs under GMP conditions for use in human clinical studies.

Here, we describe the manufacturing process overseeing the production of TBSV NPs within a small-scale laboratory setting, meant to be GMP-compliant and designed to produce quantities sufficient for toxicological pre-clinical studies and Phase 1 clinical trials in humans. All projections presented throughout the article are based on a hypothetical API dosage of 2 mg per patient/year, aimed at achieving the desired clinical outcome of tolerance induction in RA patients.

## Materials and methods

### Host plant species selection and biomass production

In the described experimental setup, *N. benthamiana* plants are cultivated indoors under controlled environmental conditions, maintaining a constant temperature of 24°C ± 3°C and relative humidity between 50% and 60%, with a photoperiod of 16 hours light and 8 hours dark. Seeds are initially sown and allowed to germinate for up to 10 days, after which the plantlets are moved into larger pots to accommodate growth and are redistributed across trays. Plants are irrigated every 2–3 days for a period of 18 days, until they reach optimal biomass. At this stage, the average leaf fresh weight (LFW) per plant is approximately 8.2 g. Five-week-old *N. benthamiana* plants are then ready for infection, initiating the manufacturing workflow, which is divided into upstream and downstream processing phases.

### Upstream process

#### Primary infection

*N. benthamiana* plants used in the process are grown under the conditions described above.

*In vitro* transcription is used to produce TBSV.pLip infectious RNAs (Grasso et al., 2013), of which 4 µg per plant are used for the manual infection of two leaves in *N. benthamiana* with the aid of an abrasive powder (Celite^®^). After 6 days, leaves displaying local and systemic infection symptoms are harvested, pooled and checked for recombinant virus RNA by reverse transcription polymerase chain reaction (RT-PCR). The collected leaves are homogenized in 1X Phosphate Buffered Saline (PBS), composed of 151 mM NaCl, 8.4 mM Na_2_HPO_4_ × 12 H_2_O, 1.86 mM NaH_2_PO_4_ × H_2_O, adjusted to pH 7.2), at a ratio of 1:10 (g LFW/mL buffer), resulting in a suspension containing virions, which is used in the next step.

#### Secondary infection

Forty microliters of sap, a solution containing infectious plant material, are used to infect one leaf, for a total of two leaves per plant, with the same rubbing procedure described above; once again, the state of the infection is monitored by phenotypical assessment, then, 6 days post infection, the leaves are harvested and homogenized with 1X PBS to obtain another infectious suspension to proceed analogously with the infection of a third batch, for the third step of the process named tertiary infection.

#### Tertiary infection

The plant biomass originating from this phase represents the starting material to be processed for final product purification. The necessary quality controls are performed during all phases, to ensure structural integrity of the virus and presence of the peptide of interest. These include RNA extraction for RT-PCR and sequencing, as well as Western blot and Coomassie staining on a native agarose gel for TBSV.p.Lip NPs quantification.

For RT-PCR, total RNA is extracted using protocols provided by the manufacturer of the TRIzol™ reagent (Invitrogen). Following RNA extraction, cDNA is synthesized, and PCR is carried out using primers TBSV_CP_For (TGCAACTGGTACGTTTGTCATATC) and TBSV_2_Back (AAGATCCAAGGACTCTGTGC). RNA extraction is the only part of the process that uses a small amount of hazardous chemicals, due to extraction with Trizol™ agent, such as chloroform and lithium chloride, but it is an essential step for quality control, and for which other commercially available extraction kits are under evaluation, to lower the environmental impact on production.

Western blot analysis is performed using a 1% agarose, 38 mM glycine gel run under native electrophoretic conditions. After addition of the 6X loading dye (for 10 ml: 6 ml glycerol 100%, 1 ml Tris-HCl 0.5 M pH 6.8 and 18 mg bromophenol blue) to the samples, they are loaded onto the agarose gel, and electrophorized at 100 V for 45 minutes. Following electrophoresis, proteins are transferred onto a nitrocellulose membrane and the presence of TBSV is detected using 1:3000 anti-TBSV primary antibody (TBSV-CO – Prime Diagnostic) and horseradish peroxidase-conjugated anti-rabbit polyclonal antibody diluted 1:3000 (Clinisciences). The signal development is obtained on washed membranes by enhanced chemiluminescence (Amersham Biosciences, Amersham, UK) and the images acquired through ChemiDoc Imaging System (Biorad). For Coomassie staining, the gel is run in the same conditions and then stained with 30 mL Quick Coomassie Stain (Clinisciences).

### Downstream process

The downstream purification process begins with the collection of the infected leaves obtained from the tertiary cycle of infection. The biomass is first weighed to determine the leaf fresh weight and then homogenized accordingly with 3 volumes of extraction buffer (sodium acetate 50 mM pH 5.3, 1% ascorbic acid) (plant-to-buffer ratio, weight in mg to volume in ml) using a steel blender, then filtered using four layers of Miracloth^®^. After this step, the homogenate undergoes a sedimentation process overnight at 4°C, to help precipitate debris and plant components. The precipitate is then consolidated with a first super-centrifuge step at 8,000 g for 15 minutes at 4°C, after which the clarified supernatant is collected and undergoes an ultra-centrifugation round at 90,000 g for 1h, 4°C. Both centrifugation steps are performed with an Avanti JXN26 Centrifuge (Beckman Coulter). The pellet obtained after ultracentrifugation is resuspended in 0.7% physiological buffer solution (NaCl in Water for Injection), centrifuged once again at 8,000g for 15 minutes at 4°C and filter sterilized: this represents the final product, which then undergoes all quality control steps, as described below. At the end of both primary and secondary infection, the product undergoes RT-PCR, sequencing and a Western blot for confirmation, respectively, of RNA and coat protein identity. After the tertiary infection, the API is produced and the following controls are performed: SDS-PAGE, Western blot, DAS-ELISA for quantification, Dynamic Light Scattering (DLS), and an LAL test as described by the manufacturer to assess endotoxin content (Gen Script).

For SDS-PAGE analysis, TBSV.pLip NPs sample is supplemented with 0.5 volume of R buffer (for 10 ml: 3 ml of Tris-HCl 0.5 M pH 6.8, 2.4 ml glycerol 100%, 1.6 ml SDS 10%, 1 ml Bromophenol blue in TE, 300 µl EDTA 15 mM, 1.7 ml H_2_O; 6.25% of 2-mercaptoethanol freshly added). After the addition of R buffer, samples are incubated at room temperature for 3 minutes and vortexed, then heated at 60°C for 1 minute, vortexed again, and kept on ice until electrophoresis performed with a SurePAGE™, Bis Tris, 12% gel (Genscript). TBSV.pLip NPs are then visualized using silver staining, following the manufacturer’s instructions (Pierce™ Silver Stain Kit, Thermo Scientific).

The Western blot is performed as described in the upstream process using a 1% agarose, 38mM glycine gel run under native electrophoretic conditions.

DAS-ELISA is used to determine TBSV.pLip NPs concentration in solution. Briefly, 150 µl of sample diluted in diluent solution (0.2% BSA - PBS-Tween 0.05% pH 7.4) are distributed in ELISA Maxisorp plates (NUNC) previously coated with 150 µl of primary antibody TBSV-Co (Prime Diagnostic) diluted 1:1000 in carbonate buffer (Na_2_CO_3_ 1.59 g/L, NaHCO_3_ 2.93 g/L pH 9.6). The presence and quantity of TBSV.pLip NPs is determined by adding 150 µl secondary alkaline phosphatase conjugated antibody TBSV-AP (Prime Diagnostic) diluted 1:1000 in diluent solution and 100 µl p-Nitrophenyl Phosphate (pNPP) substrate (Kementec). The reaction is stopped using 100 µl of 0.1 M NaOH. Plate is read using a Tecan Infinite 200 PRO plate reader at 405 nm. To quantify NPs, the ELISA test includes a calibration curve ranging from 30.02 to 0.06 nanograms of TBSV.pLip.

For DLS analysis, 0.4–0.5 mg/mL of TBSV NPs are analyzed three times using a Zetasizer instrument. Each measurement represents the mean of 12 repetitions.

### Manufacturing facility

The facility described was designed for the production of a GMP-compliant biopharmaceutical, with manufacturing volumes suitable to support Phase 1 Clinical Studies and toxicological studies. In all laboratory rooms air is filtered using High Efficiency Particulate Air (HEPA) filters, to avoid any source of contamination. Following the laboratory rooms, an isolating door grants access to the production and downstream chamber areas. All areas of production are subject to pressurization, with the three main chambers (growth, infection and downstream chambers) presenting a higher pressure than the adjacent corridors, which in turn present a higher pressure than the outside laboratories. This allows for air to flow from production towards the outside environment, thus avoiding the entry of external contaminations. Prior to entering the infection and downstream chambers, the use of proper Personal Protective Equipment (PPE) and specific dressing is mandatory, as well as removal of PPE is necessary prior to exiting. Irrigation of the plants is performed manually and to achieve optimal plant growth, the LED system has been designed to comprise three light channels: red (660 nm), blue (450 nm) and white (prevalently in the region between 500 and 600 nm). All LED have a certified degree of protection IP65, and an efficiency of 3.15 µmoles/J. Each LED is 100 cm long and four of them are meant to fit a 200 cm x 60 cm shelf, arranged for plants growth, and four shelves compose a rack, for a total capacity of 1024 plants in both the growth room and the infection room.

## Results

### Process and facility design

In Diamante SB, *N. benthamiana* is used as a self-building, single-use, biodegradable bioreactor to produce a therapeutic nanomaterial named TBSV.pLip NPs. The company facility has been carefully designed to accomplish this purpose, mindful of the active use of plant viruses in the production process. The equipment used for quality control and production is cGMP compliant. The laboratory rooms in the facility are equipped to perform all quality control analyses of the product. The current production process is controlled and monitored during working hours by working personnel. The high presence of manual operations during the production process, such as plant handling, plant-material extraction and centrifuge runs, at the moment do not allow for a more automated process, but remote monitoring is currently being implemented. Specific equipment like the -80°C freezer, used to stock material, and the general ventilation system are controlled remotely, allowing for tracking and fast intervention at any time.

**Figure 1:**
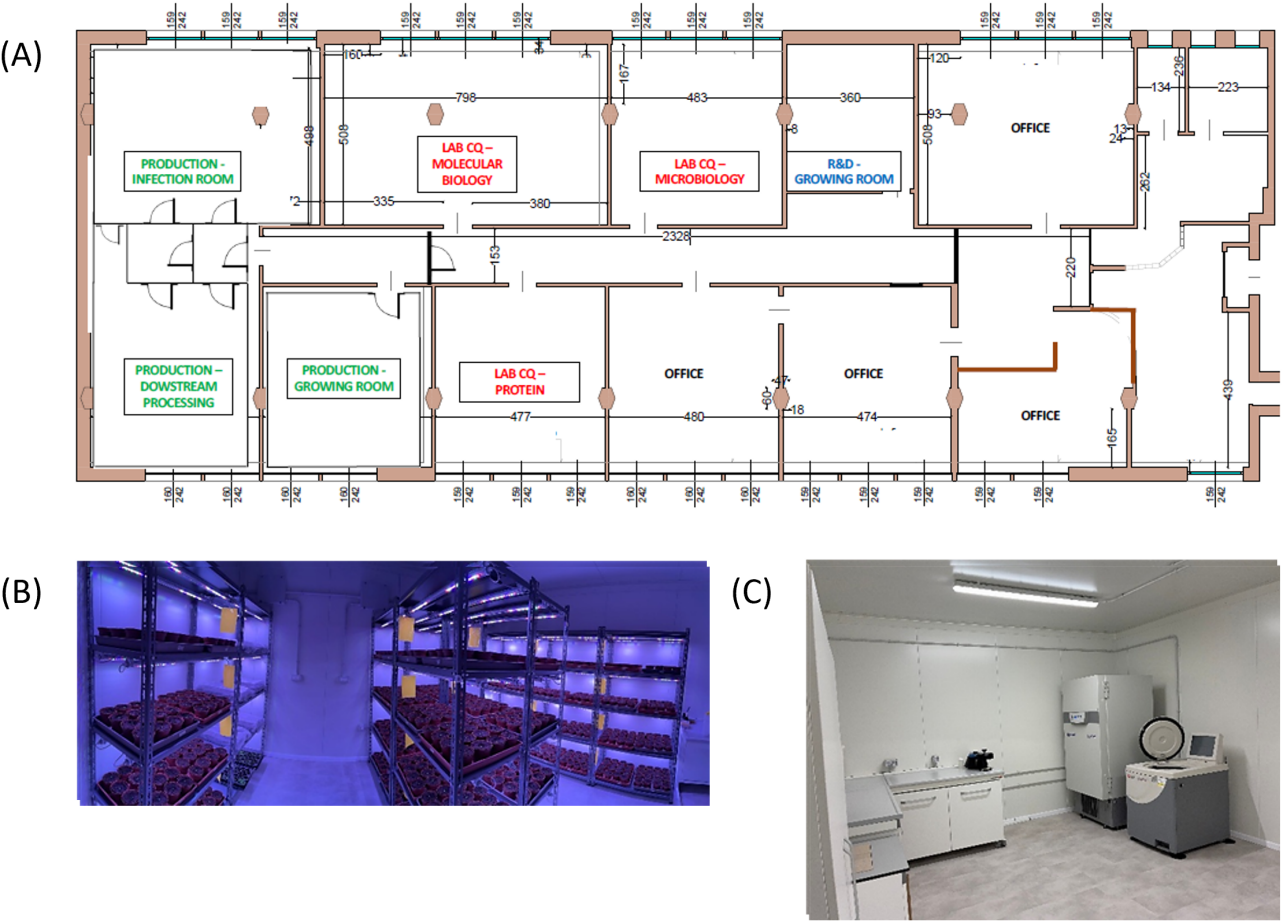
Overview of the facility. **(A)** Facility floor plan. **(B)** Production - growing room: together with the infection room, where the upstream process takes place. **(C)** Production – downstream processing dedicated room.

An overview of the facility is shown in **Figure 1**.

### Manufacturing of TBSV.pLip nanoparticles

**Figure 2:**
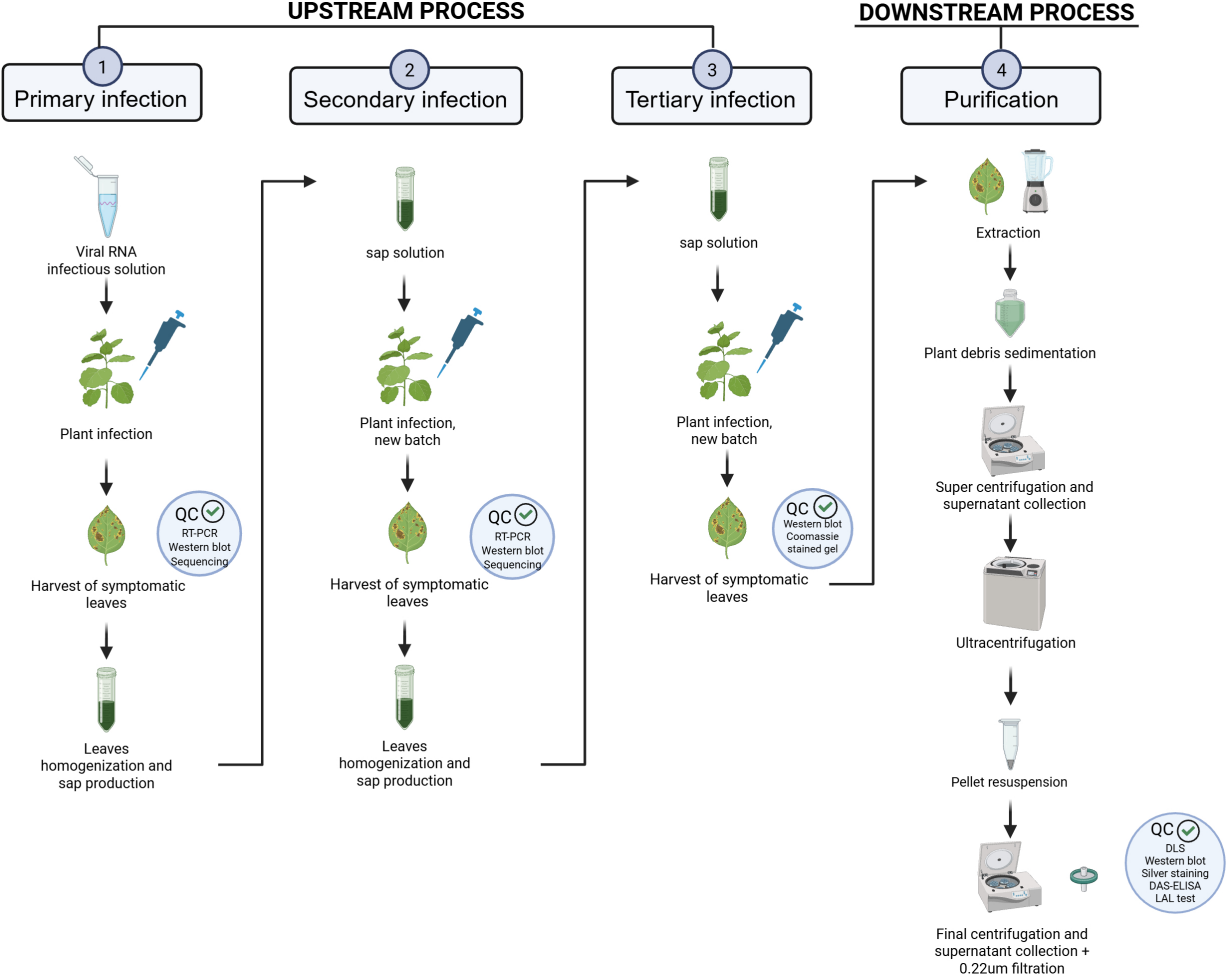
Process overview. The initial phase of the process, referred to as the upstream stage, is subdivided into three successive infection steps: primary, secondary, and tertiary. Following the tertiary infection, the product undergoes purification through the downstream processing phase.

TBSV.pLip NPs are produced using a process that can be summarized as described in **Figure 2**. The upstream process is divided into three subsequential sections, beginning with the primary infection, as described previously (Lico et al., 2021). Once symptomatic leaves are harvested, they are extracted to produce sap as starting material for a secondary infection. The same process is repeated with leaves collected from this second step, to continue with a tertiary infection, after which leaves are harvested and directly processed in the downstream phase. This last phase ends with collection of TBSV.pLip NPs. DAS-ELISA assay is used to correctly quantify the API. It has been estimated that the average total production yield for one batch of plants is 71 mg/kg. This was calculated in relation to the plants used for all the production phases, as summarized in **Table 1**.

**Table 1:** Starting material and process yields from the four phases described.

Phase	Starting material	Nr of plants infected	Yield
1	4 ug of RNA	1	1.4 g LFW
2	860 mg of SAP	107	150 g LFW
3	8.2 g of SAP	1024	1.4 kg LFW
4	1.4 Kg LFW	–	99.4 mg TBSV ± 22%

LFW. leaf fresh weight.

**Figure 3:**
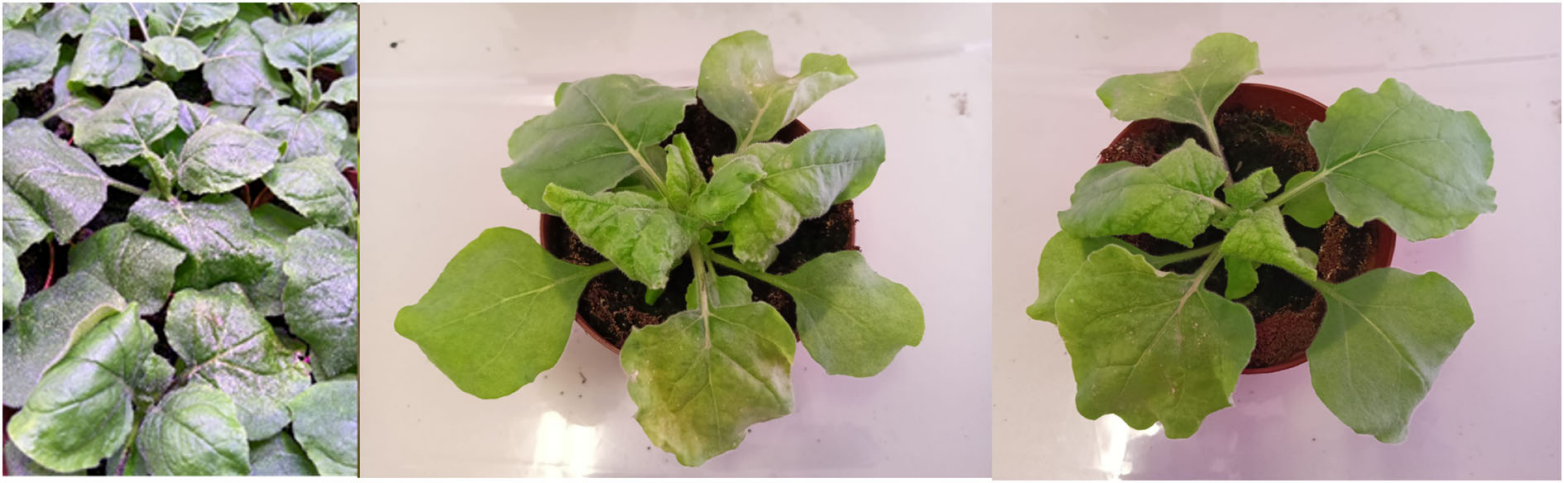
Plant infection. Example of symptomatic *N. benthamiana* plants infected with TBSV.

For each stage of the process *N. benthamiana* leaves are collected only when symptomatic. Typical symptoms of virus presence in a plant include curling of the leaves and chlorosis (**Figure 3**). It is only these leaves that are processed to obtain the final product.

**Figure 4:**
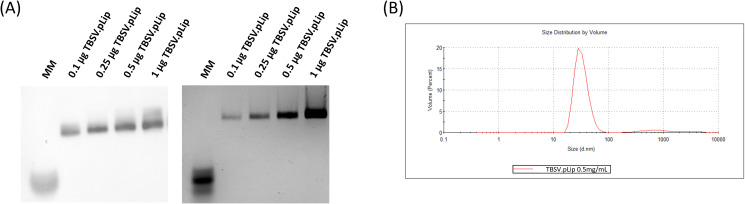
Analyticals overview. **(A)** 1% agarose gel transferred to a nitrocellulose membrane and blotted against an anti-TBSV antibody and 1% agarose gel stained with Coomassie Quick Stain show a calibration curve with increasing quantities of TBSV.pLip; **(B)** DLS profile of TBSV.pLip sample concentration 0.5 mg/mL, indicating distribution by volume: the peak indicates that most of the population is in the range of 30 nm, which is the molecular size of the virus. This graph shows the mean value obtained from three separate measurements on the same sample, each of which is the mean of 12 measurements. The standard deviation of the three measurements is 0.65.

During each step of the process, the necessary quality controls are performed to ensure NPs integrity: RT-PCR, Western blot, Coomassie stained agarose gels and sequencing are part of the upstream phase, whereas in the downstream phase, the controls comprise SDS-PAGE, Western blot, DAS-ELISA, DLS and LAL Test (**Figure 4**; **Table 2**).

**Table 2:** Quality control of the API.

Phase	Analysis	Purpose	Acceptance criteria
Upstream	RT-PCR	Amplification of the viral genome from plant extracts	Primer-specific band
	Sequencing	Peptide conformity check	<3 silent mutations
	Western blot	VNP assembly check and identification	Positive identification
	Native gel-coomassie staining	Quantification of VNPs	TBD
Downstream	DLS	VNP size and poly-dispersity index	Size 30 ± 5nm PDI: <0.3
	Western blot	VNP assembly check and identification	Positive identification
	SDS-PAGE Silver staining	Purity of the preparation	> 99%
	qDAS-ELISA	Quantification of the virus	> 0.5mg/mL
	LAL test	Quantification of endotoxin levels	< 1EU/mL

### Economic analysis of TBSV production costs

The production system in the current facility allows for the annual cultivation and processing of 13 full batches, each comprising of 1,024 plants: each batch is housed in vertically stacked racks under high-efficiency LED lights, and it grows over the course of 6 days and yields a total of 1.4 kilograms (LFW) of biomass. Due to the capacity of the available ultracentrifuge, which is the primary bottleneck in downstream processing, a maximum of 256 plants can be processed per day. As a result, each full batch is processed over four consecutive days, in sub-batches of 256 plants per day. The processing of a single sub-batch yields approximately 24.9 mg of TBSV.pLip NPs. Therefore, the total yield per full batch is 99.6 mg of TBSV.pLip NPs. Given the production of 13 batches per year, the estimated annual yield is approximately 1.3 grams of TBSV.pLip NPs. To obtain 1 gram of TBSV.pLip TBSV NPs, 10 full batches are required. The API is manufactured in *N. benthamiana* using TBSV genome as a vector of expression and exploiting the TBSV Coat Protein for the display of the peptide Liprin (pLip) on the external surface of the capsid.

**Figure 5:**
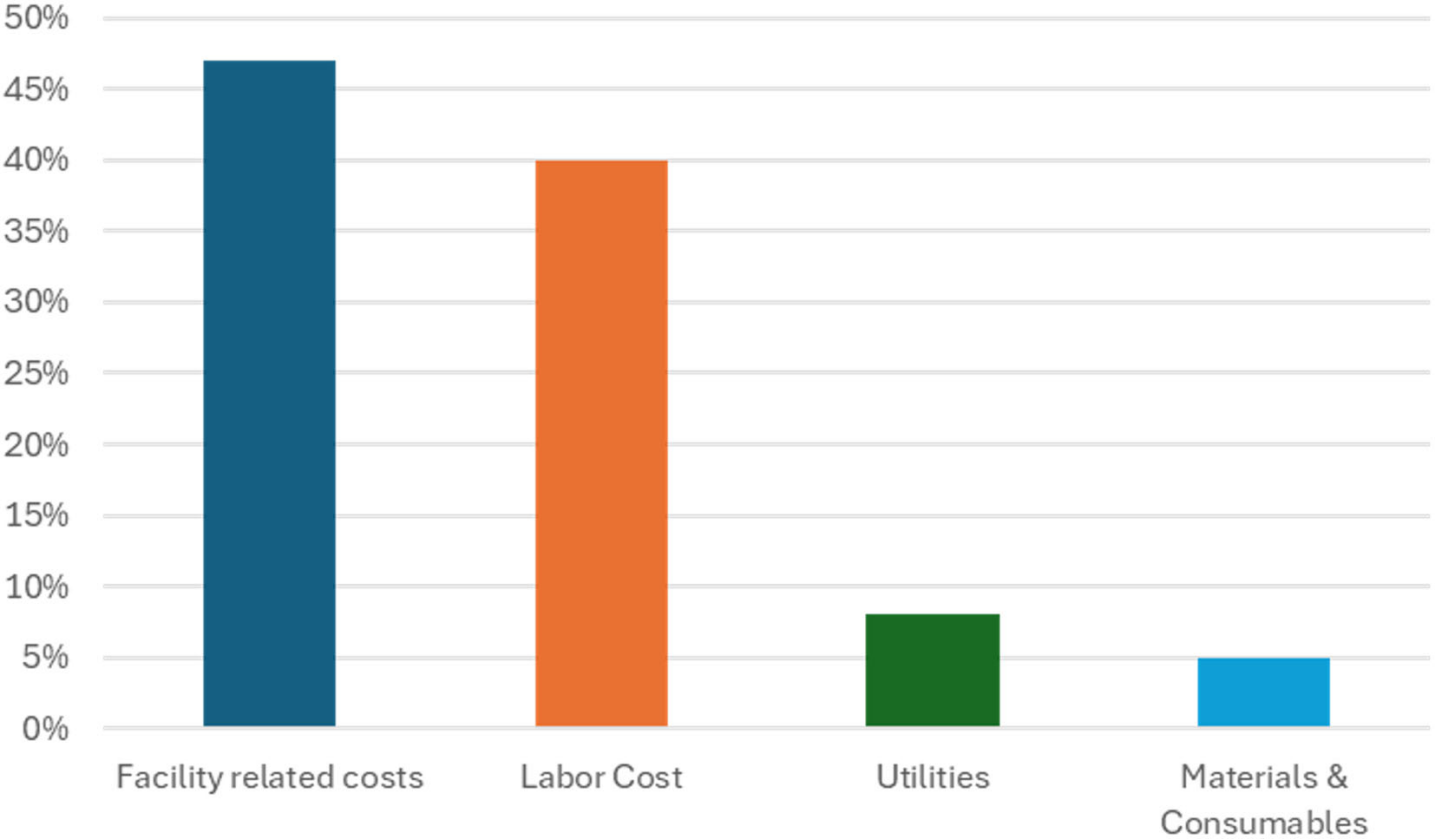
Breakdown of the percentage contribution of each cost category to the total production cost.

A comprehensive economic analysis was conducted to assess the operational expenditure (OPEX) associated with the production of 1 gram of TBSV and it is reported in **Table 3** with the corresponding graphical illustration in **Figure 5**. This analysis is based on current small-scale batch production data and includes both direct and indirect costs, while capital expenditures (CAPEX) such as facility depreciation, equipment amortization, and long-term investments are excluded from the present assessment.

**Table 3:** Breakdown of the process cost per category, including direct and indirect costs. All cost values reported here reflect the aggregate expenses incurred to produce 1 gram of TBSV, inclusive of upstream (US) and downstream (DS) operations, as well as quality control (QC) and managerial oversight.

Component	Percentage	Component	Percentage
Direct costs			
Raw Materials & Consumables	1%	Materials & Consumables	5%
QC Materials & Consumables	4%		
Production Labor Cost	14%	Labor Cost	40%
Production Manager Cost	11%		
QC Labor Cost	6%		
QC Manager Cost	9%		
Indirect costs			
Electricity	5%	Utilities	8%
Water	1%		
Heating	2%		
Rent	40%	Facility-Related Costs	47%
Building Maintenance	3%		
Cleaning Services	5%		
Total	100%	Total	100%

The results highlight that labor-related costs constitute the most significant portion of the annual operational direct cost, representing 40% of total OPEX. This includes both production labor (€ 10991.63 - 14%), QC labor (€ 5054.14 - 6%), and their respective managerial roles (€ 8462.52 - 11% for production management, € 6891.95 - 9% for QC management). These findings reflect the labor-intensive nature of the process, especially under small-scale conditions requiring high oversight and manual handling. Facility-related expenses emerged as the second major cost component, contributing € 37100.00, corresponding to 47% of total OPEX. The largest element within this category is facility rent, which alone accounts for € 31500.00 corresponding to 40%, followed by cleaning services (3600.00 € - 5%) and building maintenance (€ 2000.00 - 3%). These are fixed costs that remain constant regardless of output volume, thereby exerting a strong influence on cost per unit at limited production scales. Utilities, including electricity (€ 3938.34 - 5%), water (€ 501.38 - 1%), and heating (€ 1969.17 - 2%), together contribute 8% (€ 6408.89) of total OPEX. These costs are primarily associated with machinery operation and climate control, particularly during downstream processing steps. In contrast, materials and consumables represent only € 4276,48 corresponding to 5% of the total OPEX. This includes raw materials (€ 994.89 - 1%) and quality control reagents and consumables (€ 3281.59 - 4%). Despite being essential to the production process, these inputs have minimal economic impact compared to labor and infrastructure costs.

### Upstream *vs*. downstream cost distribution

In the upstream phase, including cultivation, infiltration, and incubation, the dominant costs are labor-related. Production labor and its associated management constitute a combined 22.6% of the total OPEX, while QC labor and materials used during in-process checks contribute an additional approximately 2%. The use of raw materials in upstream operations accounts for a modest 1% of the total operational budget. In the downstream phase, comprising tissue harvesting, clarification, ultracentrifugation, and product release testing, labor remains the primary cost driver. QC labor and QC management represent a combined 12%, reflecting the intensive resource demands of analytical validation and batch release procedures. Production labor associated with downstream extraction amounts to less than 1%, while QC materials and consumables contribute to 3%. The cost of raw materials used in this phase is negligible (<1%).

### Spatial productivity and vertical farming integration in plant-based TBSV production

**Figure 6:**
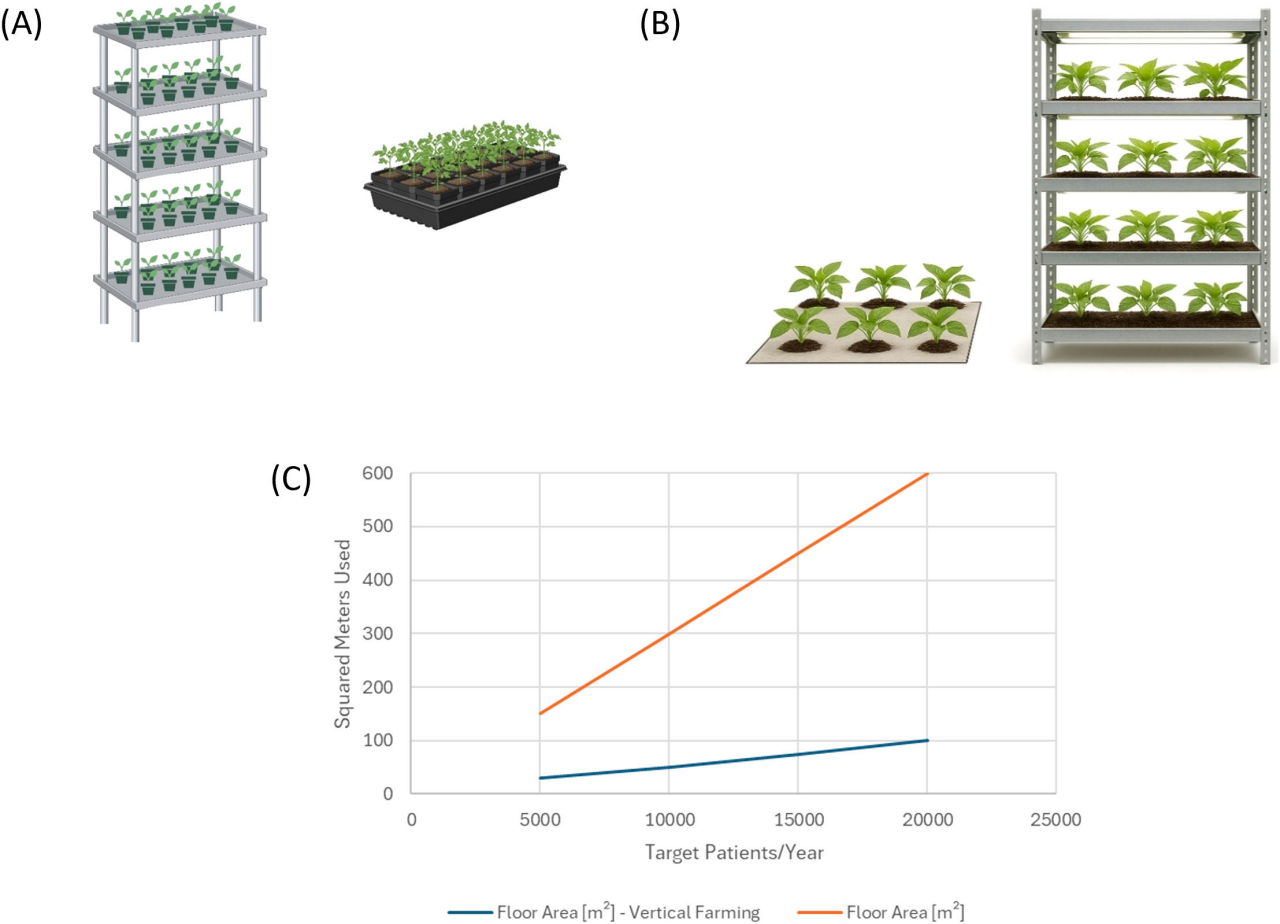
**(A, B)** The advantage of vertical farming versus traditional plant cultivation. The use of shelves allows to take advantage of the entire room area, extending cultivation in vertical layers and optimizing the space **(C)**.

An essential parameter for evaluating the scalability of plant-based production platforms is spatial productivity, commonly expressed as yield per unit area. In the current system, the TBSV.pLip NPs yield has been estimated at 6.2 mg per square meter of cultivation area per batch. Assuming 12 months of active production per year, this corresponds to an annual yield of approximately 80.6 mg per square meter per year. Assuming an annual therapeutic dose of 2 mg of TBSV.pLip per patient (a dosage that remains to be validated for clinical studies based on the delivery of 4 injections of 500µg of TBSV.pLip per patient), this production rate allows one square meter of cultivation space to support treatment for approximately 40 patients per year. To meet a therapeutic demand of 5,000 patients per year, an estimated 125 square meters of cultivation area would be required. This requirement increases proportionally with treatment demand, reaching 1,250 square meters and 25,000 square meters for 50,000 and 1,000,000 patients, respectively (**Table 4**). To optimize land use efficiency and reduce the physical footprint of the production facility, a vertical farming system should be adopted (**Figure 6**). By incorporating five levels of vertically stacked shelving, the required floor area is effectively reduced by a factor of five. Accordingly, the floor area needed to treat 5,000, 50,000, and 1,000,000 patients per year is reduced to 25 m², 250 m², and 5,000 m², respectively (**Table 4**).

**Table 4:** scalability of TBSV.pLip NPs production by using vertical farming.

Target patients/year	Required floor area	Floor area - vertical farming (5 levels)
5,000	125 m²	25 m²
50,000	1,250 m²	250 m²
1,000,000	25,000 m²	5,000 m²

## Materials hazardness and environmental impact

The workflow described includes the use of a limited number of hazardous chemicals, such as Trizol^®^, chloroform, hydrochloric acid, and lithium chloride. Importantly, these substances are utilized in minute quantities—typically in microliters per batch—and their contribution to the overall environmental footprint is negligible. Strict adherence to laboratory safety and waste management protocols further mitigates their risk.

One potential concern unique to our system is the use of TBSV as a vector. While TBSV is a plant-specific virus with no known risk to humans or animals, its presence in biomass waste could raise biosafety considerations, particularly in open-field or large-scale settings. We recognize this challenge and are currently investigating strategies to inactivate the virus post-harvest, ensuring that the final biomass is free of viable viral particles before disposal or downstream use. In summary, the combination of (i) low-volume reagent usage, (ii) elimination of persistent organic solvents, (iii) the biodegradable nature of plant material, and (iv) ongoing viral inactivation efforts, reinforces the environmental advantages of our production method.

## Discussion

Despite the recent closure of Medicago following the withdrawal of investment by its parent company, the global landscape of plant-based biotechnology companies remains dynamic. Notable examples include Protalix Therapeutics (Israel), with two marketed products—Elelyso^®^ and Elfabrio^®^; BioApp (South Korea), developer of the HERBAVAC™ pig vaccine; KBio (USA), focused on various recombinant proteins; Eleva (Germany), advancing a moss-derived therapeutic for Fabry disease; InVitria (USA), producing technical-grade proteins; ORF Genetics (Iceland), supplying growth factors and cytokines for cosmetics and research; Agrenvec (Spain) and Baiya Phytopharm (Thailand), both targeting applications in cultivated meat and cosmetics; and CapeBio (South Africa), developing medical diagnostic solutions.

In this vibrant and dynamic scenario, Diamante SB, founded in 2016 as a spin-off of the University of Verona with support from European public funding programs such as Bio-based innovation for sustainable goods and services with the project PharmaFactory and the EIC accelerator, has successfully transitioned from an academic setting to an industrial reality. The company is focusing on the development of a platform for autoimmune disease treatment with the first therapeutic application in Rheumatoid Arthritis. Here we report a basic techno-economic analysis from laboratory data collected in the first year of work in the new lab-setting meant to obtain GMP certification disclosing the production process of the therapeutic TBSV.pLip NPs meant for Rheumatoid Arthritis treatment.

Plant-based biomanufacturing of therapeutic viral proteins represents a relatively novel platform with few commercial-scale facilities currently in operation. However, it provides several advantages, including linear production scalability, simplified upstream processes, shorter time to market, and the potential for reduced capital and operational expenditures.

The facility described here annually produces 1.3 g of TBSV.pLip NPs, the API, from 18.2 kg LFW *N. benthamiana* leaves. The plant, selected for its productivity and host of TBSV infection, is inherently more sustainable than traditional production platforms, largely due to its biodegradable nature and its low-impact cultivation requirements. In general, plants serve as renewable, carbon-sequestering bioreactors, and their post-harvest waste can often be composted or disposed of with minimal environmental burden (Buyel, 2019).

Furthermore, *N. benthamiana* is familiar to European Medical Agency (EMA) and Food and Drug Administration (FDA) thus facilitating its acceptance in regulation-compliant manufacturing (Streatfield and Howard, 2003; McCormick et al., 2008; Bendandi et al., 2010; Tusé, 2011; Plant Viral Vectors for Delivery by Agrobacterium, 2013).

13 batches are seeded and grown annually, with one batch reaching harvest every 28 days. Expression rate of 355 mg of TBSV.pLip NPs per kilogram of biomass (fresh weight) and a downstream recovery of 20% give a yield of 71 mg of TBSV.pLip NPs per kilogram of harvested biomass. This corresponds to 6.2 mg of TBSV.pLip NPs per square meter of cultivation area per batch, the combination of this approach with spatial modularity offered by vertical farming solutions offers a clear advantage for scalable models, enabling increased productivity per unit footprint and facilitating expansion without the need for large horizontal land allocations. When combined with the inherent flexibility of plant-based systems, vertical farming provides a strategically efficient pathway for transitioning toward larger-scale, economically sustainable production of biopharmaceuticals such as TBSV.pLip NPs.

The economic feasibility of TBSV production at small scale was conducted by analyzing direct operating expenditures (OPEX), which include labor, materials, and utilities in both upstream and downstream processes. Capital expenditures (CAPEX) were excluded to focus on recurrent, process-dependent costs. As detailed in the Results section, labor and facility-related costs dominated the cost structure, accounting for 40% and 47% of total OPEX, respectively, while materials and consumables contributed only 5%.

Based on the annual yield of approximately 1.3 grams, the direct OPEX per mg of TBSV.pLip was estimated at €27.61. This cost reflects the fixed-intensive nature of the process and highlights the potential for improvement through economies of scale. Increasing batch numbers or optimizing facility use would reduce per-unit costs significantly.

Moreover, the labor-heavy profile suggests that selective automation, especially in routine handling and monitoring, could reduce manual workload, improve consistency, and support future scale-up.

However, it is important to highlight that a critical point in the described production of TBSV.pLip NPs is the potential environmental risk associated with the particles, which are currently treated as infectious material. This classification significantly impacts disposal costs and logistics. At present, viral inactivation strategies are being evaluated, which will require confirmation through preclinical studies to ensure therapeutic efficacy. Previous work in oncology (Mao et al., 2021) has shown that when using virus nanoparticles genetic material inactivation can negatively affect therapeutic performance, making this validation step essential.

Together, these strategies represent key opportunities to enhance the economic sustainability of TBSV-based production, a platform that may be explored for diverse tolerance induction applications simply changing the peptide displayed by virus nanoparticles.

Finally, it is important to note that in addition to the conventional cost of goods, Life Cycle Assessment accounting for the costs of the environmental footprint of manufacturing should be addressed, even in the field of biopharmaceuticals.

When comparing our process with standard methods used for the chemical synthesis of peptides, the core therapeutic agent, the footprint of the plant-based upstream process is negligible when compared to the volumes of organic solvents used in traditional solid-phase peptide synthesis (SPPS). In contrast, SPPS is well-documented for its environmental burden, primarily due to its reliance on toxic, non-biodegradable solvents (e.g., dimethylformamide, dichloromethane) and repetitive washing steps, which generate significant chemical waste. The cumulative impact of these solvents not only raises health and safety concerns but also requires energy-intensive waste treatment and solvent recovery systems.

This approach represents a meaningful step toward greener, safer, and more scalable biomanufacturing when compared with traditional peptide synthesis platforms and it could be used as a platform for the production of nanomaterials meant for tolerance induction in the framework of autoimmune diseases.

## Conclusion

This study demonstrates the potential of a plant-based platform for nanoparticle production, with a disruptive impact on therapies for autoimmune diseases. Although the described facility and processes still require optimization in certain areas, they can be implemented within a compact space and, with a relatively modest investment, to effectively support Phase I clinical trials.

## Data Availability

The raw data supporting the conclusions of this article will be made available by the authors, without undue reservation.
